# Allicin and Digestive System Cancers: From Chemical Structure to Its Therapeutic Opportunities

**DOI:** 10.3389/fonc.2021.650256

**Published:** 2021-04-27

**Authors:** Mahshad Sarvizadeh, Omid Hasanpour, Zari Naderi Ghale-Noie, Samaneh Mollazadeh, Mohammad Rezaei, Hossein Pourghadamyari, Mohammadjaber Masoud Khooy, Michael Aschner, Haroon Khan, Nima Rezaei, Layla Shojaie, Hamed Mirzaei

**Affiliations:** ^1^Nutrition and Endocrine Research Centre, Research Institute for Endocrine Sciences, Shahid Beheshti University of Medical Sciences, Tehran, Iran; ^2^School of Paramedicine, Kashan University of Medical Sciences, Kashan, Iran; ^3^Department of Medical Genetics, Mashhad University of Medical Sciences, Mashhad, Iran; ^4^Natural Products and Medicinal Plants Research Center, North Khorasan University of Medical Sciences, Bojnurd, Iran; ^5^Department of Diabetes, Obesity and Metabolism, Cell Science Research Center, Royan Institute for Stem Cell Biology and Technology, ACECR, Tehran, Iran; ^6^Department of Clinical Biochemistry, Afzalipour School of Medicine, Kerman University of Medical Sciences, Kerman, Iran; ^7^Department of Agronomy, Science and Research Branch, Ahvaz Islamic Azad University, Ahvaz, Iran; ^8^Department of Molecular Pharmacology, Albert Einstein College of Medicine, Bronx, NY, United States; ^9^Department of Pharmacy, Abdul Wali Khan University, Mardan, Pakistan; ^10^Department of Medical Immunology, School of Medicine, Tehran University of Medical Sciences, Tehran, Iran; ^11^Research Center for Immunodeficiencies, Children's Medical Center, Tehran University of Medical Sciences, Tehran, Iran; ^12^Network of Immunity in Infection, Malignancy and Autoimmunity (NIIMA), Universal Scientific Education and Research Network (USERN), Tehran, Iran; ^13^Department of Medicine, Research Center for Liver Diseases, Keck School of Medicine, University of Southern California, Los Angeles, CA, United States; ^14^Research Center for Biochemistry and Nutrition in Metabolic Diseases, Institute for Basic Sciences, Kashan University of Medical Sciences, Kashan, Iran

**Keywords:** allicin, gastrointenstinal cancer, therapy, chemical structure, natural compounds

## Abstract

Digestive system cancer tumors are one of the major causes of cancer-related fatalities; the vast majority of them are colorectal or gastric malignancies. Epidemiological evidence confirmed that allium-containing food, such as garlic, reduces the risk of developing malignancies. Among all compounds in garlic, allicin has been most researched, as it contains sulfur and produces many second degradation compounds, such as sulfur dioxide, diallyl sulfide (DAS), diallyl trisulfide (DATS), and diallyl disulfide (DADS) in the presence of enzymatic reactions in gastric juice. These substances have shown anti-inflammatory, antidiabetic, antihypertensive, antifungal, antiviral, antibacterial, and anticancer efficacy, including gastrointestinal (GI) cancers, leukemia, and skin cancers. Herein, we summarize the therapeutic potential of allicin in the treatment of GI cancers.

## Introduction

Cancer is a widespread disease, leading to the death of 7.9 million patients annually, accounting for 13% of all deaths globally (1, 2). It has been estimated that cancer mortality rates will continue to rise to ~12 million in 2030 (3). In 1970, developed countries faced higher numbers of reported cancers (around 85%), compared with developing countries (only 15%) (4). Nonetheless, around 70% of cancer cases have arisen in developing countries (5). The risk of developing chronic medical conditions has increased, and this trend will be 5-fold greater by 2030 (6). Among all chronic illnesses, cancer has been considered as the second main cause of death, even ahead of cardiovascular diseases (7). The second most prevalent reason for cancer death has been reported to be gastrointestinal (GI) cancer (8). In GI malignancies, the involvement of related organs, including colon, intestine, and esophagus, have been reported (9). It is noteworthy, several genetic variations in oncogenes, tumor suppressors, and mismatch repair genes may result in GI carcinogenesis (10). One significant reason for the pathogenesis of GI cancer is the imbalance between apoptosis and cellular proliferation (11). Several external and internal parameters, including genetic factors, life style (such as alcohol consumption and obesity), and infection (such as *Helicobacter pylori*) have a role in GI cancer pathogenesis (12).

The main health problems caused by gastrointestinal cancers impose significant burdens on healthcare systems (13). GI prognosis varies in affected people depending on the progression of illness at the time of diagnosis. It is worth mentioning that early diagnosis of GI cancer is important for the improvement of outcomes of the patients. The present treatments include surgery, radiation, and chemotherapy, with several components, including cisplatin, mitomycin, and docetaxel injection (14).

The organic sulfur-containing organosulfur compounds (OSCs) have been shown to possess antioxidant, anti-inflammatory, and anticancerous features (15, 16). Studies in animals have shown that OSCs have the ability to decrease colon cancer risk by inducing mitotic arrest and apoptosis (17–19). These compounds are abundant in asparagus, garlic, onion, and cruciferous vegetables. Recently, given the high content of flavonoids and OSCs, such as allicin in garlic, scientists have focused on the efficacy of these compounds in cancer therapy (20).

Garlic is a plant commonly consumed as a dietary additive across the world (21). Garlic has a notable and useful impact due to OSCs like allicin (22). Allicin or diallyl thiosulfinate are types of OSCs (1% of dye weight of garlic) (23) that are generally present in garlic, *Allium sativum* L., with numerous bioactivities. Allicin can be produced from tissue damage of the non-proteinogenic amino acid S-allylcysteine sulfoxide (alliin), which is catalyzed by alliinase enzyme (24). It should be noted that allicin has antioxidant activity (25) and inhibits the growth of both bacteria and fungi (26, 27).

## Allicin Chemical Structure and Biological Actions in Cancer

**Figure 1:**
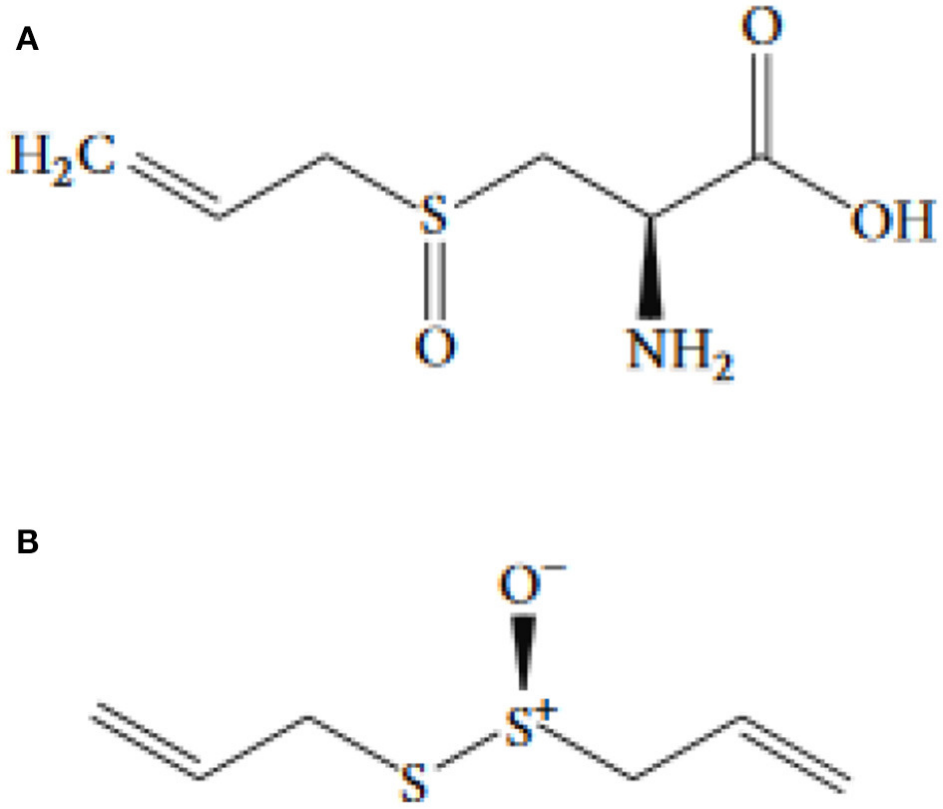
The chemical structure of **(A)** allicin and **(B)** alliin.

Pioneering studies (28) have proposed that two compounds provide the flavor of garlic distillates, namely diallyl trisulfide (DATS) and diallyl disulfide (DADS). Cavallito and Bailey (29) introduced allicin as the most bioactive compound in garlic (29, 30). Figure 1 shows the allicin structure. Chopping or cutting the garlic cloves results in allinase activation (29–32). In the cloves, ~70% of all thiosulfinates after mechanical cutting and crushing is represented by allicin (33–35).

**Figure 2:**
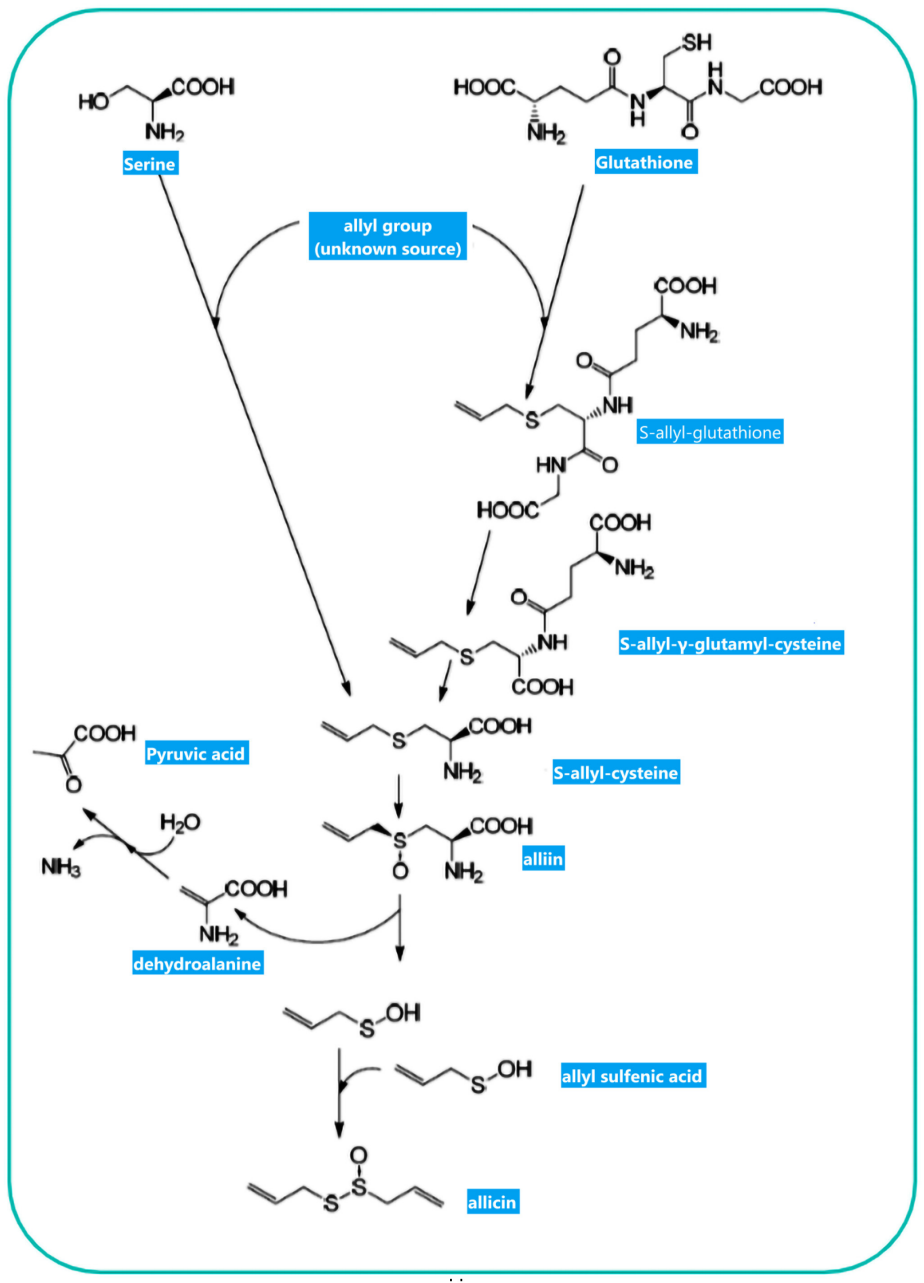
Allicin biosynthesis: Two biosynthetic routes result in S-allylcysteine. The observation of 14C-labeled S-allylcysteine following feeding plants with 14C-labeled serine and applying several alkyl mercaptans led Granroth to posit that serine is one potential substrate for S-allylcysteine biosynthesis. Another pathway resulted from GSH to S-allylcysteine. The allyl-group source has yet to be determined. S-allylcysteine, after oxidization, is converted to alliin as an “inactive” precursor of allicin. Enzymatic hydrolysis of alliin produces allyl sulfenic acid, which can be condensed spontaneously to allicin. This figure adapted from Borlinghaus et al. (24).

The non-proteinogenic amino acid called alliin (S-allyl-l-cysteine sulfoxide) is the precursor of allicin (36). Alliinase is an enzyme that hydrolyses alliin and other S-alkyl-l-cysteine sulfoxides. Alliin hydrolysis results in dehydroalanine and allyl sulfenic acid production. One allicin molecule is generated secondary to the spontaneous condensation of two allyl sulfenic acid (24, 37). Alliin is present in ramsons (*Allium ursinum*) and garlic (*Allium sativum*) (38). Interestingly, onion (*Allium cepa*) cannot produce alliin, but synthesize its isomer isoalliin [trans-(+)-S-(1-propenyl)-l-cysteine sulfoxide] (39). The biological route of alliin synthesis has yet to be clarified. Two feasible biosynthetic pathways have relied on radioactive labeling studies (Figure 2) (39). Following the chemical synthesis of allicin, *in vitro* protocols have been published for its enzymatic production (40, 41). The substrate alliin is extracted from garlic gloves or synthesized from cysteine by allyl bromide alkylation followed by hydrogen peroxide oxidation (42). In general, allicin purification is incomplete, as related compounds such as ajoene, polysulfane, and vinyldithiine are present due to high reactivity and reduced thermal stability of allicin (43). Pure allicin is stable in dilute aqueous solutions at −70°C preparations for several years (no loss for 2 years) (44).

In 2004, Miron et al. (41) described a simple method for the preparation of 3(H)-labeled allicin. Alliin is the key sulfur composition in either raw or powdered garlic. Cloves of garlic have about 8 g/kg of alliin. Furthermore, the dehydration reaction, absent loss of ingredients, results in producing 20–25 mg/g of alliin in the powdered garlic. The powdered garlic possesses maximally 10 g/kg of alliin, representing reduced alliin content following the dehydration process. Crushed fresh garlic contains around 37 mg/g of allicin (41). Diallyl thiosulfinate (allicin), as the main constituent of solvent extracted garlic, is dehydrated, resulting in the formation of two isomeric disulfides *via* the transformed rearrangement. After a day, diallyl monosulfide, DADS, DATS, and sulfur dioxide are produced as the main results of this reaction (45). Allyl and methyl sulfides are the main components of commercial garlic oils. The allicin is degraded and converted rapidly into vinyldithiins, ajoenes, and DADS. This process has been influenced by various conditions, including temperature, pH, and concentration (46). Allicin can form DADS (a lipid-soluble oligosulfide) in the presence of heated aqueous media (47). The distinctive smell of garlic cloves, which can affect a person's breath, is due to an allyl mercaptan compound. The complexation of allicin with cysteine (Cys) in the blood results in allyl mercaptan generation (48). The key pathways of medicinal (non-enteral and non-topical) function of DADS or allicin are allyl mercaptan, or a subsequent metabolite (48). The allicin content has been measured by the high-performance liquid chromatography (HPLC) method in 24 botanical characteristics of the garlic ecotypes in Iran (49). The content of allicin ranges from 0.16 to 13.0 mg/g. The correlation between the content of allicin and ecological condition has been denied.

In 1992, Lawson and Hughes (50) examined the impact of several factors, including post-acidification neutralization, pH value, temperature, and the time on the thiosulfinate released from garlic cloves and powder. In the pH values of 4.5–5.0 (the optimum value), all dipropenyl thiosulfinates like allicin were formed. No thiosulfinate was present at pH < 3.6. Another study by Yu and Wu (51) focused on the pH effects on the flavor compositions formed from allicin. Their study revealed that the maximum levels of 2-vinyl-1,3-dithiin and 3-vinyl-1,2-dithiin (isomeric cyclic ingredients) are obtained at pH 6.5, whereas DAS, methyl allyl disulfide, DADS, and DATS were formed maximally at pH 9.0.

Allinase acts on alliin to form allicin. Allicin can be rapidly metabolized into S-allylmercaptocysteine, diallyl sulfide (DAS), S-allylcysteine, DADS, DATS, vinyl dithiines, and ajoene (52–56). The enhanced allicin formation was accompanied by elevation in pH (4.0–6.0) of the macerating medium (57). Furthermore, a rise in pH level (4.0–6.0) contributed to upward rate of allicin decomposition (57). Eating or biting raw garlic leads to feeling the sensation of burning and pain on the tongue or lips.

**Figure 3:**
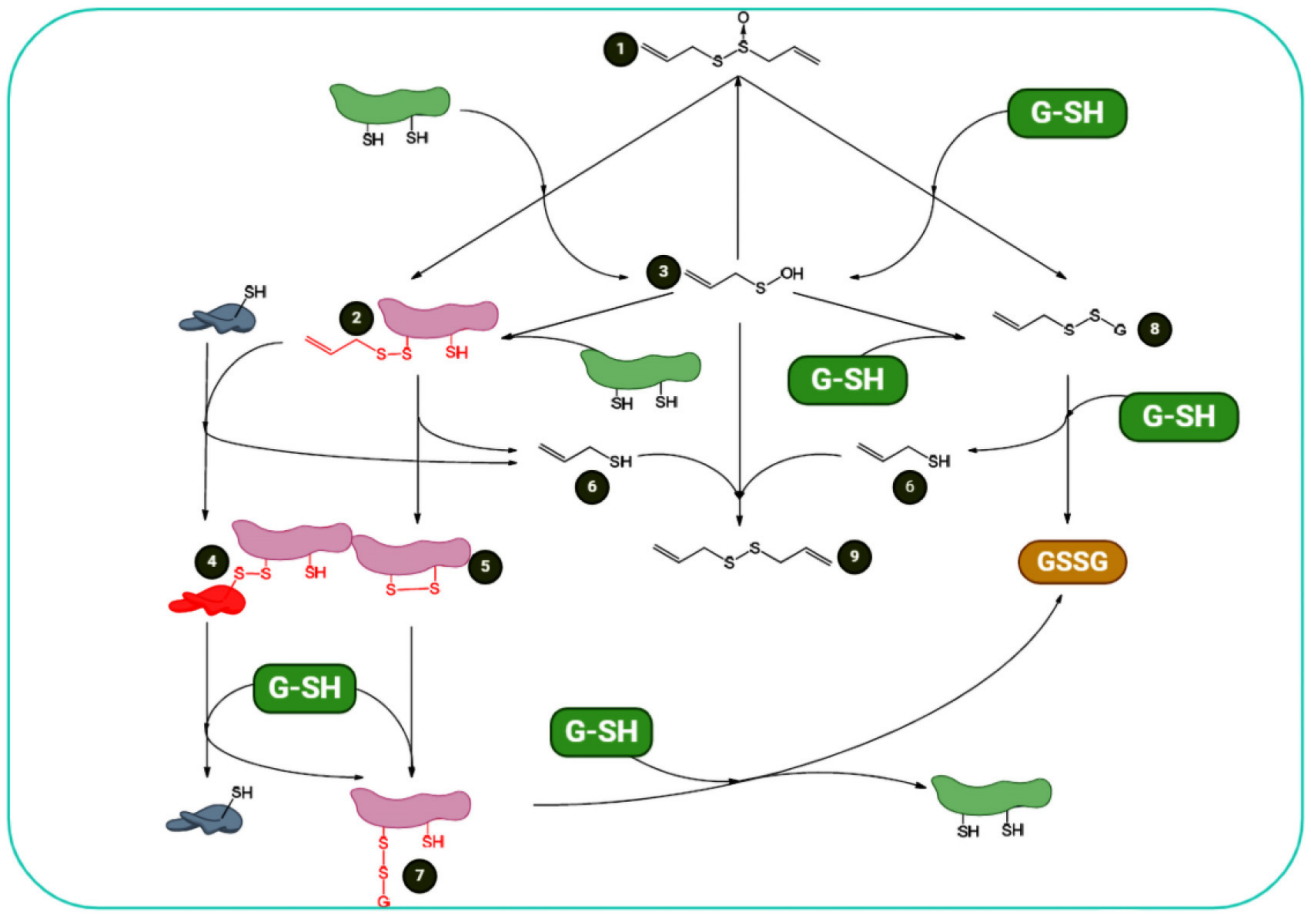
Summary of allicin and cellular thiols: redox chemistry of allicin shows that (1) it can react with cellular thiols such as Cys-containing proteins and GSH. Its reaction with proteins results in formation of S-allyl-mercapto-proteins (2) and allyl sulfenic acid (3). S-allyl-mercapto-proteins can react with other proteins *via* disulfide bond-stabilized complexes (4) or generate intramolecular disulfide bonds (5). Both reactions result in the elimination of allyl mercaptan (6). Protein disulfide bonds are reducible by cellular GSH, resulting in S-glutathionyl-mercapto-proteins (7). To omit the glutathionyl residues from the proteins, another GSH is required. Additionally, allicin reacts with GSH. This interaction results in S-allyl-mercapto- GSH (8) and allyl sulfenic acid (3). S-allyl-mercapto-GSH can undergo a thiol/disulfide exchange reaction with an additional GSH to form allyl mercaptan and glutathione disulfide (GSSG) (6). Allyl sulfenic acid (3), formed upon direct reactions of thiols and allicin, can react with proteins to form S-allyl-mercapto-proteins (2), with GSH to form S-allyl-mercapto-GSH (8), with allyl mercaptan (6) to DADS (9), or with additional allyl sulfenic acid (3) to produce allicin *de novo*. This figure adapted from Borlinghaus et al. (24).

As a reactive sulfur species (RSS) with oxidizing characteristics, allicin can oxidize thiols in cells, e.g., Cys residues and glutathione (GSH) (58). A greater pool of oxidized GSH will result in a higher potential of cellular redox. The protein thiol oxidation may lead to alterations in protein structure, for instance, by the formation of a disulfide bond (for details, see Figure 3). Redox-stimulated structural alterations in proteins may result in gain- or loss-of-function. These effects are established for the plant protein NPR1, a critical protein in pathogen-induced immunity. These properties are also observed in yeast (*Saccharomyces cerevisiae*) for YAP1 as a redox-sensitive transcription factor organizing the response of oxidative stress (59, 60). YAP1 is analogous to the redox modulated mammalian Nrf2/Keap1/ARE system (61).

Allicin and DADS stimulate the sensory neurons that are sensitive to allyl isothiocyanate and activate capsaicin-sensitive perivascular sensory nerve ends, which can cause vasodilation (62). Miron et al. (41) revealed that TRPA1 (62) and TRPV1 [both are the temperature-activated ion channels belonging to the family transient receptor potential (TRP)] were activated by raw but not baked garlic. These thermo-TRPs have been shown in pain-sensing neurons with a role in innervating the mouth. It was reported that TRP1 and TRPV1 activation was due to unstable component of allicin, a reason of pungency of garlic. During storage the amount of allicin in organic solvents can be affected by temperature. Its loss has been reported very low at temperatures of −16 and 6°C when comparing with storing at room condition (43). When stored in ethyl acetate at room temperature, its amount reduced to 10% within 3 days. Nevertheless, it took nearly 13 days in methanol. Consequently, the allicin is more stable in methanol than ethyl acetate. In a study by Iberl et al. (63), it has been shown that allicin had stability in solvents due to hydrogen bonding. Likewise, in 1992 Lawson and Gardner (64) focused on several characteristics of garlic productions, including stability, bioavailability, and composition (two non-sulfur and 14 sulfur ingredients). Under simulated GI and *in vivo* conditions, the availability of allyl thiosulfinates (mainly allicin) has been examined. At a temperature of −80°C, the stability of allyl thiosulfinates of blended fresh garlic was minimally for 2 years. The enteric-coated garlic tablets can release the dissolution of thiosulfinates more than 95%. It is worth mentioning that the allyl thiosulfinate bioavailability in these tablets and breath was equal to the crushed fresh garlic. At ambient temperature, the stability of S-allylcystein lasted 12 months. In order to measure the stability, thiosulfinates of blended garlic with no chosen condiment and at the temperature of 4°C, were evaluated. During 12 days, the amount of thiosulfinates did not significantly decrease. Moreover, drying the garlic at 60°C could not affect the alliin although after drying, only a 4% yield loss was reported for allicin, dimethyl thiosulfinates, and allyl methyl thiosulfinates. Nevertheless, around 75% yield loss for each 1-propenyl thiosulfinates demonstrated that the drying step resulted in destroying a large amount of isoalliin (50).

The function of the immune system and cancer are closely related. In a primary implantation study (1960), Dipaolo et al. incubated the mouse-tumor explants in allicin prior to implantation in healthy mice. In order to compare the control with allicin-treated explants, the tumor growth has been investigated in both groups. The mice with allicin-incubated tumor explants did not reveal further explant growth (65). In molecular studies, the allicin impact has been evaluated on the malignant cancer cells. Clearly the anticancer effect of allicin was owing to the apoptosis induction (66). This is a reason for the death of cell, in both manners of caspase-dependent (67) or caspase-independent (68). In addition to caspase activity, apoptosis-inducing factor (AIF) is another leading factor, conducting the DNA-laddering apoptosis, which triggers cell death caused by allicin.

In a study by Bat-Chen et al. (23), it has been shown that nuclear factor erythroid 2 (NF-E2)-related factor 2 (Nrf2) has a function in allicin-induced apoptosis. In 2008, Loboda et al. suggested the “Janus face” of Nrf2 (69), it means that the Nrf2 has both antiapoptotic and proapoptotic role. As an antiapoptotic factor which is mostly reported, it can regulate the Bcl-2 family (antiapoptotic proteins) expression like Bcl-xL and Bcl-2 (70, 71). Furthermore, in the immune cells, allicin affects the extracellular signal-regulated kinase 1/2 (ERK1/2). Thus, these kinases can play a substantial role in allicin-induced apoptosis (26, 66).

**Figure 4:**
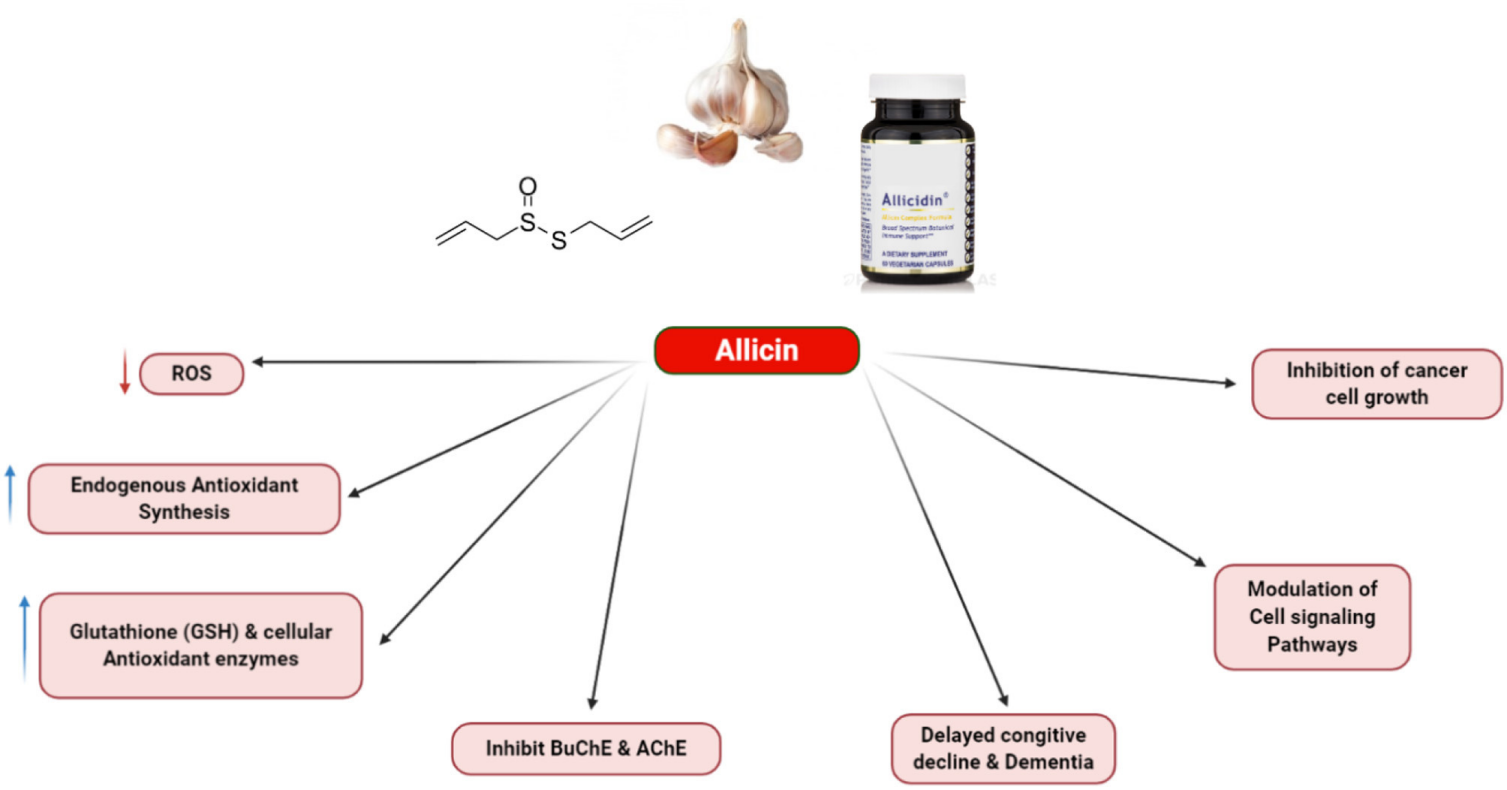
Various biological action of allicin.

Although this section briefly explains the allicin effects on the cancerous cells, multiple allicin-targeted key factors are also summarized. The instability of allicin is one problem, which prevents its chemical applications. In the circulatory system, the accessible thiols can react with allicin and decompose to other compounds. Therefore, this is unlikely to use it as the pharmaceutical factor at the moment. Nevertheless, the possible applications of garlic in the nutriceutical field, having health benefits have been frequently reported in numerous medical areas, especially for the prevention of cancer and therapeutic approach. The way to address the problem with stability is to couple the alliinase to a delivery system and also to supply the stable substrate of alliin, which can generate allicin at certain epitope positions under *in situ* conditions. Accordingly, antibody-mediated cancer cell/alliinase coupling has been considered a promising approach to prevent various cancers (72). Figure 4 shows some biological actions of allicin.

Protein kinase activated by adenosine monophosphate (AMP) is a conserved energy sensor with a critical function in modulating lipid and protein metabolism (73). AMP-activated protein kinase (AMPK)-p38-peroxisome proliferator-activated receptor gamma coactivator (PGC)-1α axis regulates energy homeostasis and sustains the survival of cancer cells in glucose-limiting scenarios. As a molecular switch, AMPK increases glycolysis by phosphofructokinase (PFK2) activation and promotes mitochondrial metabolism of non-glucose carbon sources by maintaining the level of cellular ATP. Chaube et al. (74) showed that AMPK could facilitate oxidative metabolism by promoting mitochondrial biogenesis and idative phosphorylation (OXPHOS) capacity by controlling the expression of PGC-1α *via* p38 mitogen-activated protein kinase (MAPK) activation (74, 75). It has been shown that aged garlic extract (AGE) stimulates the AMPK activation in adipose tissues, liver, and gastrocnemius muscles in a type-2 diabetes model (76). In addition, AGE has been shown to mediate AMPK phosphorylation in the liver in an atherosclerosis mouse model (77). To date, these studies have shown that AGE increased AMPK activity in several animals and tissue models, though the mechanisms of AGE-mediated AMPK activation have yet to be deciphered. It has been well-established that AMPK-mediated phosphorylation can activate tuberous sclerosis complex 2 (TSC2), which downregulates the mechanistic target of rapamycin (mTOR) (78). Chu et al. (79) reported that allicin reduced the PI3K/mTOR signaling pathway, cytoplasmic p53, and the Bcl-2 level and elevated Beclin-1 signaling pathways AMPK/TSC2 expression in Hep G2 cells.

## Allicin as Potential Therapeutic Option in Digestive System Cancers

### Allicin as Potential Therapeutic Option in Colon Cancer

Experiments in human colon carcinoma cell lines demonstrated that chemopreventive activities of allicin are associated with maintenance of mitochondrial membrane, regulation of intracellular redox, and cell proliferation (15). In addition, allicin leads to G2/M arrest, mitochondrial membrane potential disruption, and intracellular GSH modulation (80). A transient reduction in intracellular GSH content has been shown in response to allicin (53). While the mechanism and function of OSCs have not been completely identified, evidence corroborates that several targets including c-Jun N-terminal kinase (JNK)/MAPK, p38, and ERK1/2 expression along with Phase 2 detoxifying enzyme gene are involved (81). A leucine zipper transcription factor referred to as Nrf2 is a regulator of Phase 2 detoxifying enzymes and antioxidant proteins (82). Nrf2 is involved in several cytoprotective aspects including anticarcinogenicity, anti-inflammation, and neuroprotection. Nrf2 normally is cytoplasmic agent. In order to activate the Nrf2, an interaction should occur between Nrf2 and Kelch-like ECH associating protein 1 (Keap1) as actin-binding protein, and then it is degraded rapidly *via* ubiquitin–proteasome pathway. Nrf2, in turn, dissociates from Keap1 when the Nrf2–Keap1 complex is targeted by electrophilic insults or reactive oxygen species (ROS) signals. Consequently, the stabilized Nrf2 undergoes nuclear translocation to trans-activate the target genes (83). The target genes of Nrf2 include GSH-generating enzymes, antioxidant proteins, and Phase 2 detoxifying enzymes. Accordingly, the chemopreventive activities associated with allicin can affect the Nrf2 (23).

In order to isolate active allicin, Bat-Chen et al. (23) introduced a simple and novel approach. They found allicin is a compound with stable, amenable features, which was soluble in water (23). Moreover, they evaluated how allicin affected the division and proliferation of colon cancer cell lines (Caco-2, HT-29, LS174T, and HCT-116) and investigated the essential pathways. It has been reported that the allicin treatment leads to HCT-116 apoptosis as revealed by higher level of hypodiploid DNA, enhanced levels of bax as well as upward trend of mitochondria-to-cytosol release of cytochrome c, and Bcl-2 decline. Moreover, allicin can induce Nrf2 translocation to HCT-116 cell nucleus. When Nrf2 was knocked down *via* siRNAs, it considerably had effect on the allicin potential to inhibit the HCT-116 proliferation. In colon cancer cells, Nrf2 is suggested to be a key mediator that contributes to allicin-induced apoptotic death (23).

A specific characteristic of allicin is rapid metabolization in the blood samples of humans under *in vitro* conditions (43) and of rats under *in vivo* conditions (84). Notably, it is debatable whether distant targets from the GI system (such as endometrial or mammary tissues) can be directly affected by allicin. However, the high allicin potential in influencing various metabolic routes with regard to its rapid metabolism (85), suggest that the wide range of beneficial health impacts can be attributed to the allicin metabolites and not allicin itself. Indeed, several such metabolites act as anticancer agents. Some studies addressed these chemical compositions, such as S-allylmercaptocysteine (54), DAS, DADS, and DATS (55, 86), ajoene (52, 87), and S-allylcysteine (56), which are different according to the agent and the experimental model. Recent studies (88) have shown that the human liver has the ability to produce allicin from DADS through liver microsomes. Hence, although allicin is rapidly removed from the bloodstream, its metabolites are capable of reforming it through the interconversion process and, consequently, act intracellularly. The exact mechanisms, whereby allicin has an influence on different cellular systems, are unclear. Allicin also has a role in scavenging hydroxyl radicals (89, 90) and inhibiting the production of superoxide through the granulocytes activated by phorbol ester in human beings (91). Furthermore, allicin can affect numerous cellular proteins by reacting with thiol-containing compounds (85, 89, 92). For instance, in adenocarcinoma cell line, it has been proposed that the allicin selectively blocks the conversion of the GSH-dependent prostaglandin H2 to the prostaglandin E2 isomerase. In contrast, the results of another study propose that an intense increase in fructose 1,6-bisphosphatase activity of chicken liver has been observed in the presence of allicin (93). Because crude garlic extracts contain numerous active substances, including OSCs with varying stability and biological activity, detailed mechanistic studies of the effects produced by individual chemically defined garlic components on tumor cell proliferation are of great importance. There have been some reports over allicin compounds, which affect various signaling pathways and cellular metabolic including: protein phosphorylation, mitogenic machinery, calcium transport, and hormone metabolism, and responsiveness (94). Herein, a question regarding the allicin remains unanswered, whether it is crucial for efficient impacts of garlic extract generally, or for anticancer activities particularly (43, 95). Additionally, since a stable and purified allicin preparation is available (96), comparing the preparation activity with crude garlic aqueous extract is possible.

*In vitro* model study determined whether the antiproliferative effect reported for garlic is due to pure allicin (53). Allicin had an inhibitory role in the proliferation of endometrial (Ishikawa), colon (HT-29) and human mammary (MCF-7) cancer cells [half maximal inhibitory concentration (IC50) = 10–25 μM]. In three experiments on primary human fibroblast line, two tests exhibited the same reaction to allicin (IC50 = 16–40 μM), although the result of third line almost was not affected by this ingredient. Furthermore, the similar potency, which has been observed in mere allicin and garlic powder extracted water with equivalent concentrations of allicin, proposing that, allicin has an antiproliferative role in the extract. The cell accumulation in the G2/M and G0/G1 cell cycle phases (MCF-7 cells) leads to growth inhibition, and not *via* a marked elevation in cell death. The allicin is a contributing factor to transient reduction in the level of GSH, according to cell type, the kinetics and magnitude of which were significantly different. It is worth noting that there was a significant correlation (*r* = 0.75) between the extent of reduction in GSH levels and allicin-induced growth inhibition. All of the above-mentioned data suggest that the allicin is a major antiproliferative factor in the water-soluble garlic formulations, probably due to transiently intracellular GSH depletion due to allicin activity (53).

Signal transducer and activator of transcription-3 (STAT-3) regulates various processes, including cytokine signaling pathways, cell proliferation, and apoptosis through phosphorylation by multiple ligands (97). The activation of STAT-3 is an initiator to transcript the target genes, such as Bcl-xL, Mcl-1, p21, and Bcl-2, acting in cell proliferation and survival (98). In human cancer cells, the activation of STAT-3 contributes to persistent STAT-3 target gene activation, which can stimulate some pathways like angiogenesis, apoptosis prevention, and cell growth, thereby driving tumorigenesis. Furthermore, it has been suggested as a key factor explaining the fate of intestinal epithelial cells during colitis and colitis-associated colorectal cancer (CAC) (98). The inhibition of STAT-3 signaling leads to induction of apoptosis in CAC cells *via* the mitochondria and particularly Bcl-2 modulation (99).

In a colorectal cancer mouse model, Li et al. (100) examined the allicin effect on azoxymethane/dextran sodium sulfate (AOM/DSS) and investigated the underlying possible mechanism. They reported that the allicin could have *in vivo* inhibitory effect on the tumorigenesis of colon by the AOM/DSS in mice. Moreover, a study revealed that allicin leads to apoptosis induction and suppressing the HCT-116 cell proliferation and survival under *in vitro* conditions. In addition, several studies on the molecular mechanism have focused on the STAT3 signaling suppression. Therefore, these data support the role of allicin as a potential favorable supplement for human colorectal cancer (CRC) (100).

In another study, allicin effects were tested on mouse fibroblast (3T3), human umbilical vein endothelial cell (HUVEC), human lung epithelium carcinoma (A549), human colon carcinoma (HT29), and human breast cancer (MCF7) cells (101). They performed the standard methyl thiazol tetrazolium (MTT) test to analyze the allicin toxic effects on the cell viability and 3H-thymidine incorporation in the cell proliferation. To measure the reactive species and GSH pool, they used monobromobimane and 2′,7′-dichlorofluoresceine-diacetate, respectively. In order to estimate apoptosis, the YO-PRO-1 iodide staining method was performed. Allicin lessened the cell viability and proliferation dose-dependently. In the bimane test, cells treated with allicin showed a reduction in fluorescence, which was probably evidence of GSH oxidation. Different allicin sensitivity was seen for the tested cell lines in terms of the GSH oxidation, cell viability, and proliferation. Among the studied cells, the MCF-7 and 3T3 cell lines revealed a higher rate of apoptosis in comparison with other cell lines. Based on the data, sensitivity and responses to allicin can be different in various mammalian cell lines (101).

There is evidence that allicin inhibits cancer progression *via* some mechanisms (102), including cell cycle arrest (80, 103), induction of apoptosis (66, 67, 103–106), the histone acetylation induction (107), and the angiogenesis suppression (108). Accordingly, apoptosis induction mediated *via* allicin has been also displayed on some human colonic cancerous cells (HT-29 and Caco-2 cell lines) (23, 67, 68). Also, it was confirmed that incubation of HT-29 cells together with garlic extract enriched by thiosulfinate within 24 h could provoke the apoptosis induction in the cells. Moreover, allicin can be considered as a promiscuous agent because of having other significant pharmacological affects, including antimicrobial and cardiovascular efficacies (109). However, promiscuity as versatility might be a benefit for cancerous chemotherapeutic factors due to the complex tumor pathogenesis, including defects in various signaling pathways and checkpoints. Moreover, allicin affects different signal transduction pathways, which suggests that combining this lyophilized garlic extract with mechanistically individual chemotherapeutic drugs could have beneficial effects. In fact, combinatorial therapy with natural compounds could pave the promising way in cancer therapies (110–112). Additionally, this garlic extract due to its pharmacological safety has been offered to be applied lonely for cancer prevention and in addition with chemotherapy agents for cancer treatment (113).

Perez-Ortiz et al. (104) provided evidence for co-adjuvant regimes in treating colorectal tumors by introducing consumption of *Allium sativum* extract enriched by thiosulfinate combined with chemotherapy drugs [5-fluorouracil (5-FU) or oxaliplatin] on CRC cell viability (Caco-2 and HT-29). Furthermore, they presented a decline in economic points and monotherapy dosage, which can be achieved by this novel combined therapy. The thiosulfinate-enriched garlic extract increased the cytotoxic effects of oxaliplatin and 5-FU (500 μM) on HT-29 and Caco-2 cells more effectively compared to standard oxaliplatin and 5-FU drugs (114). Table 1 shows the therapeutic effects of allicin on GI cancers.

**Table 1:** Therapeutic effects of allicin on GI cancers.

**Type of allicin**	**Type of cancer**	**Dose (s)**	**Effects**	**Model**	**Cell line**	**References**
Allicin	Colon	3 and 6 μg/ml	Down-regulated the mRNA expression level of VEGF, uPAR, and HPA	*In vitro*	LoVo	(115)
Allicin	Colon	4 and 8 mg/L	Showed Antiproliferation properties and enhanced the cytotoxicity of CPT-11	*In vitro*	LoVo	(116)
Allicin	Colon	1–50 μg/ml for 24, 48, and 72 h	Through modulating Nrf2, Induced apoptosis and increased the expression of Bcl-2 and release of cytochrome *c*	*In vitro*	LS174T, HT-29, Caco-2, and HCT-116	(23)
Allicin	Colon	10–25 μM	Inhibited tumor cell growth	*In vitro*	HT-29	(53)
Allicin	Colorectal	Mice model: 48 mg/kg to achieve 5 g/day; HCT-116 cells: 25 μM for 24 h	Prevents tumorigenesis *via* inhibiting the STAT3 signaling pathway activation	*In vivo and in vitro*	HCT-116	(100)
Garlic juice and synthetic allicin	Colon	Up to 1.2 mM	Decreased cell proliferation and viability	*In vitro*	HT29	(101)
Allicin	Colon	2.5, 5, 10, 25, 50, 75, 100, and 200 μg/ml	Promoted the effects of 5-FU and oxaliplatin against cancer cells	*In vitro*	Caco-2 and HT-29	(114)
Garlic extract supplemented with garlic powder	Colon	30, 100, 300, and 100 μg/ml	Showed a dose-dependent manner of tumor cell growth inhibition	*In vitro*	Caco-2	(91)
Allicin	Pancreatic	10 mg/kg	Increased CD4+T, CD8+T, NK cell, and serum IFN-γ	*In vivo*	–	(117)
*In situ* generated allicin	Pancreatic	Alliin (20–200 μM)	Increased caspase-3 and p21 expression, DNA fragmentation, and cell cycle arrest	*In vitro*	MIA PaCa-2	(103)
Allicin and MT100	Pancreatic	20, 50, and 200 μM	Cancer cells showed lower chemoresistance to allicin and MT100	*In vitro*	AsPC-1, BxPC-3, Capan-1, Panc-1, and KPC	(118)
Allicin	Gastric	15–120 μg/ml for 72 h	Promoted release of cytochrome c, expression of 3, −8, and −9 and activation Bax and fas	*In vitro*	SGC-7901	(106)
Allicin	Gastric	0.1, 0.05, and 0.016 mg/ml	stimulated apoptosis and suppressed telomerase activity	*In vitro*	SGC-7901	(119)
Allicin	Gastric	3, 6, and 12 mg/L	Inhibited cell proliferation and induced apoptosis	*In vitro*	SGC-7901	(120)
Allicin	Gastric	3, 6, 9, and 12 μg/ml	Induced cell cycle arrest and up-regulated p21WAF1 and p16INK4 genes	*In vitro*	MGC-803 and SGC-7901	(121)
Allicin	Gastric	0.1, 1, and 10 μg/ml	*Via* modulating cleaved caspase-3 and p38, enhanced apoptosis	*In vitro*	BGC-823, MGC-803, and SGC-7901	(122)
Allicin	Gastric	NA	Increased Bax and Fas expression and decreased Bcl-2 expression level	*Human*		(123)
Ajoene analogs	Esophageal	10 μM for 16 h	Inhibited cell proliferation, induced cell cycle arrest, and caspase-3 activation	*In vitro*	WHCO1	(124)
Allicin	Hepatocellular	5 mg/kg/day, every 2 days for 3 weeks *in vivo*; 0, 1, 2, 4, 8, 10, 16, 20, 32, 40, and 64 μg/mL for SK-Hep-1; 0, 1.25, 2.5, 5, 10, 20, 40, 80, and 160 μ μg/mL for BEL-7402	Promoted caspase-3 and PARP, and down-regulated Bcl-2	*In vivo* and *in vitro*	SK-Hep-1 and BEL-7402	(125)
Allicin	Hepatocellular	0, 15, 20, 25, 35, 40, and 50 μM	Decreased MMP and Bcl-2, and increased Bax, AIF, Endo G, caspase-3,−8, and−9	*In vitro*	Hep 3Band Hep G2	(126)
Allicin (synthesized)	Hepatocellular	35 μM for 0.5, 1, 3, 6, and 12 h	Induced p53-mediated autophagy, decreased p53, the PI3K/mTOR signaling, and Bcl-2. Increased the expression of AMPK/TSC2 and Beclin-1	*In vitro*	Hep G2	(79)
Hepatic-targeted polybutylcyanoacrylate nanoparticles of diallyl trisulfide	Hepatocellular	NA	Decreased PCNA and Bcl-2 proteins	*In vivo and in vitro*	HepG2	(127)

### Allicin as Potential Therapeutic Option in Hepatocellular Carcinoma

For several decades, allicin has been applied clinically because of its different roles, such as antimicrobial, anti-inflammatory, cardiovascular protective, and immunity boosting effects (128–130). Earlier studies have found that allicin not only inhibits the growth of cancerous cell but also induces cell apoptosis in different tumors amongst are gastric carcinoma, glioblastoma, breast cancer, and hepatocellular carcinoma (HCC) (131, 132). Notably, allicin increases the sensitivity of CRC cells to chemotherapeutic agents (CPT-11) *in vitro* (116). However, the impact of allicin on chemosensitivity efficacy *in vivo* and probable molecular mechanism for the HCC treatment is still unknown.

The main challenges, which limit chemotherapeutic efficacy in HCC, are hepatic dysfunction and drug resistance (125). It has been shown that allicin has been an ideal choice to increase the effects of chemotherapy agents in HCC because of its hepatoprotective and anticancer impacts. Confirmatory results by Zou et al. (125) highlighted that allicin improved cytotoxic effects of 5-FU in HCC cells as a promising chemotherapy regimen. *In vivo* experiment in nude mice showed that the combination of allicin (every 2 days for 21 days; 5 mg/kg/day) and 5-FU (5 consecutive days; 20 mg/kg/day) dramatically inhibited the HCC proliferation and revealed highly apoptotic impacts in HCC xenograft tumor model in comparison to 5-FU alone. Furthermore, allicin and 5-FU combination therapy enhanced the ROS level in the cell, activated caspase-3 and poly(ADP-ribose) polymerase (PARP), lessened the mitochondrial membrane potential (ΔΨm), and decreased Bcl-2 expression in HCC cells in comparison to treated cells with DMSO, allicin, and 5-FU alone. In addition, antioxidant N-acetylcysteine (NAC) as ROS inhibitor could block enhancement of active PARP and caspase-3 and reduced the HCC cell hypersensitivity to the 5-FU created by allicin. In conclusion, for the first time, this research depicted that allicin had a synergistic effect with 5-FU in the HCC cell sensitization to the induced cell death *via* the mitochondrial pathway mediated by the ROS (125).

P53 is a tumor suppressor protein and takes part in multiple biological processes, such as carcinogenesis, mutation, exogenous or endogenous injury, and several cell signaling transduction, which regulate cell death and viability (133). Some reports have indicated that the p53 protein level controls the various death process, such as apoptosis (133), autophagy (134), and necrosis (135). Chu et al. (126) understood that the allicin significantly activated caspase-dependent and -independent apoptotic pathways in human hepatoma Hep 3B cells (p53 knocked down cells) by ROS upregulation. In the earlier study, Chu et al. similarly confirmed that allicin activated cell death *via* autophagy mechanism mediated by p53 in human hepatoma Hep G2 [p53(wild type)] cells. Collectively, these findings not only proposed allicin-stimulated cell death in HCC cells *via* different mechanism (autophagy or apoptosis), but also promised a new supplementary gene-based treatment approach to overcome the apoptosis resistance in tumor cells (126). As mentioned before, allicin induces cell death due to the autophagolysosome establishment in Hep G2 cell line, which was evidenced by enhancement of monodansylcadaverine expression (autofluorescent drug accumulating in autophagolysosome). Also, mitochondria destruction could be happened in a time-dependent manner by LC3-II colocalization (autophagy biomarker) and MitoTrackerRed fluorescent dye (mitochondria tracker, Waltham, MA, USA). Allicin enhanced the expression of Bad, Beclin 1 (BECN1), TSC complex subunit 2 (TSC2), phosphorylated-AMPK, and Atg7. In contrast, it reduced the amounts of cytoplasmic p53, phosphatidylinositol 3-kinase/mTOR (target of rapamycin in mammals), and Bcl-xL and p-Bcl-2 in the Hep G2 cells. In detail p53-mediated autophagy *via* allicin in liver malignant cells was through TSC2 and phosphorylated-AMPK signaling pathway activation, mTOR inhibition and suppression of cytoplasmic p53 apoptotic pathways. Furthermore, allicin could not regulate the levels of caspases (3, 8, 9) in Hep G2 cells and stimulated caspase-dependent apoptosis. Allicin-induced cell death in HepG2 cells was facilitated by mitochondrial pathway of death through a decrease of ΔΨm and mitochondrial depolarization. Additionally, the 3-methyladenine 3-MA, inhibitor of autophagy pretreatment could prevent the dotty-cluster of LC3-II–FITC and block autophagy vesicles and mitochondria colocalization. Also, 3-MA suppressed the allicin-stimulated upregulation of Beclin-1, TSC2, Atg7, and GRP78; however; it induced caspase-3 levels. Therefore, garlic-derived allicin controls autophagy *via* induction of several potential strategies, which makes it a considerable potential chemopreventive factor for liver cancer therapy (79).

The garlic-isolated allicin and other OSCs contain allylthio group in their structure. Allylthio class is recognized as a pharmacophore with different biological activities (136). Based on the study, different K-compounds (derivatives of 3-alkoxy-6-allylthiopyridazine) were produced, and their bioactivities were examined in the animals. According to the results, different derivatives not only possessed proper hepatoprotective activities on aflatoxin B1-treated rats and carbon tetrachloride-treated mice, but also exerted chemopreventive actions in rat hepatocarcinoma cells. The other novel pyridazine derivatives (sulfur-substituted compounds) in which the 3- alkoxy-6-allylthiopyridazine was replaced by sulfur (S) and the oxygen site was at the 3-position, were tested *in vitro*. Among them, Thio-K6, presented higher presented higher chemopreventive activity than K6 in hepatocarcinoma cells (SK-Hep-1) (136). Taken together, an important issue in the biological activities of garlic-derived OSCs might be related to the number of sulfurs.

### Allicin as Potential Therapeutic Option in Gastric Cancer

Allicin decreased the viability of SGC-7901 cells (human gastric adenocarcinoma cell line) dose-dependently and time-dependently, which was evidenced with the help of the MTT assay. Furthermore, allicin decreased the cell viability through apoptosis induction in cancerous cells, confirmed by Rh123 and propidium iodide staining, transmission electron microscopy, alternations in the mitochondrial membrane potential, and the Annexin V/FITC assay. Following allicin treatment in tumor cells, cytochrome c discharged from mitochondria and co-expression of bax and fas upregulated caspase-3, -8, and -9 levels. These molecular events were evaluated by the Western blot assay, immunocytochemistry, and quantitative reverse transcription PCR (Q-RT-PCR). Allicin treatment co-activated extrinsic and intrinsic apoptotic routes (respectively, Fas/FasL-mediated and mitochondrial pathways) in SGC-7901 tumor cells. Allicin should be further evaluated as a great potential agent in cancer management (preventive or therapeutic factor) in the control of gastric malignancies and other types of tumors (106).

Allicin can result in G_2_/M cell cycle arrest followed by cell apoptosis induction. The cause of allicin-induced apoptosis is associated with reduced telomerase activity dose- and time-dependently. By degradation of telomerase activity, telomere becomes shortened and mitotic process is arrested, all of which results in apoptosis (137). The Bcl-2 (antiapoptotic agent) affected the modulation of telomerase activity. The Bcl-2 overexpression causes a substantial increase in the telomerase activity level. Nevertheless, down-expression of Bcl-2 decreases telomerase activity (138–140). Molecularly, the mechanism by which allicin reduced Bcl-2 expression was *via* secondary messengers system, especially cyclic AMP and PKC followed by Fas and Bax upregulation, and concurrently Bcl-2 downregulation (141). Nonetheless, the modulation of telomerase activity is a complicated process (119).

Sun et al. (119) treated gastric malignant SGC-7901 cells with allicin to investigate the impact of this compound on both apoptosis and telomerase activity compared to AZT (3′-Azido-3′-deoxythymidine). The MTT assay showed that allicin at different concentrations (0.1, 0.05, and 0.016 mg/ml) could significantly inhibit SGC-7901 cells after 48 h compared to the control. However, microscopic images did not show any atypical morphologic alternations in the cells after allicin treatment at 0.016 mg/ml concentration for 24 h. Flow cytometry (FCM) data revealed that the allicin could induce the cell apoptosis non-linearly and dose-dependently and enhanced the cell population in the G2)/M phase more effectively than the control group. TRAP-PCR-ELISA method displayed that allicin could shorten and prevent telomerase activity dose-dependently and time-dependently in gastric cancer cell line (SGC-7901) much better than AZT (119).

Accordingly, Tao et al. (120) evaluated the allicin inhibitory effect on SGC-7901 cells and its related pathway. The findings indicated that the cell growth in the control group was logarithmically normal; however, the growth of cell inhibited in the experimental group was treated with allicin in a concentration-dependent trend. Furthermore, differences in apoptotic degree were considerably different following the allicin treatment with different increased concentrations on SGC7901 cells. FCM outcomes exhibited that different allicin concentrations at 24 and 48 h changed and regulated the SGC7901cell cycle. In detail, the proportion of G0/G1 phase cells reduced to some extent, the population of G2/M phase cells apparently enhanced, whereas the number of S phase cells altered little. Based on the findings, the anticancer effect of allicin is higher than other chemotherapeutic agents with relatively less toxicity and side effects (120).

Previous studies on other tumor cells indicate that allicin may meaningfully activate apoptosis in mouse melanoma, prostate cancer (LNCaP), and human gastric adenocarcinoma (SGC-790l) cell lines (53, 79). Additionally, the immune system may be induced by the allicin to discharge more active factors, enhancing antitumor effects of allicin and preventing tumor growth (53, 68). Unlike common chemotherapy drugs, allicin does not have toxic impacts on the body and may release multiple cytokines and increase immune resistance (142, 143).

**Figure 5:**
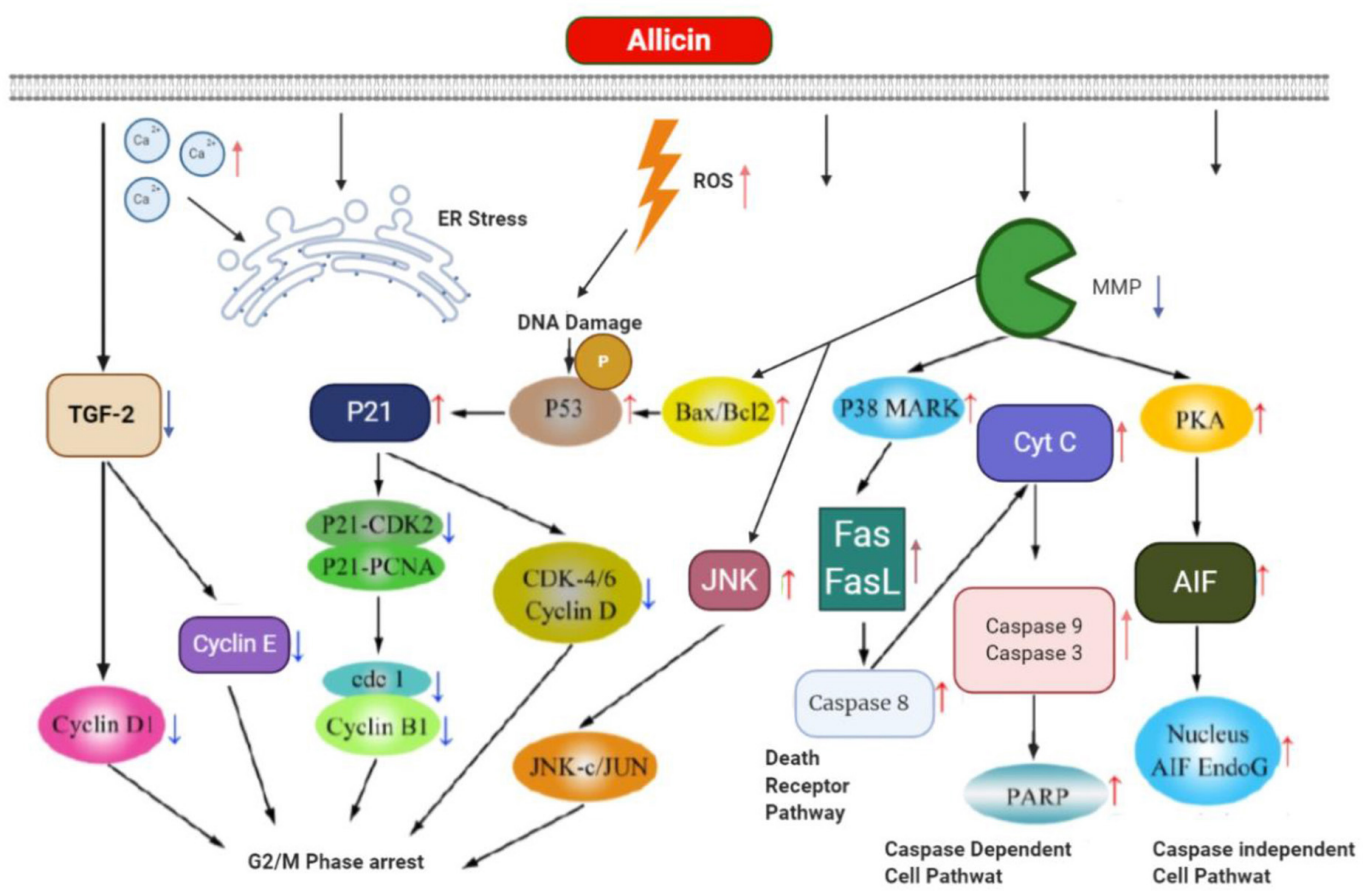
The apoptosis and its related mechanism mediated by allicin in gastric cancer cell.

Zhang et al. (122) explored the allicin inhibitory role on the MGC-803, human gastric carcinoma cell, and the related probable mechanisms. To do so, allicin effects on the cells were evaluated through measuring apoptosis by the Hoechst staining, cell viability by the MTT assay, and the expression of apoptosis-related proteins and also apoptosis mechanisms by Western blot technique. The findings demonstrated a significant increase in the apoptosis rate of MGC-803 *via* allicin time-dependently and dose-dependently. Furthermore, the rate of apoptosis and cleaved caspase 3 expression increased in allicin-treated MGC-803, and also allicin upregulated p38 protein levels. These results are in a favor of preventative role of allicin on MGC-803 cells mediated by suppression of the cell growth and apoptosis induction (122). Figure 5 shows the apoptosis and its related mechanism, mediated by allicin in gastric cancer cell.

Allicin can reduce the expression of TGF-2 and its receptor after entering directly into gastric cancer cell followed by not only downexpression of cyclinD1, cyclinE, and cyclin-dependent kinase (CDK), but also causing DNA damage and generating ROS to produce p21 and phospho-p53 (Ser15). Then p21 suppressed the CDK-4/6/cyclinD complex, P21-PCNA, P21-CDK2, and subsequently reduced cdk1/cyclinB1 complex for G2/M phase cell cycle arrest. Allicin increases the levels of Bax (proapoptotic protein), Bcl-2 (antiapoptotic protein), and JNK through reduction in outer mitochondrial membrane potential (MMP. In addition to apoptosis induction, Bax/Bcl-2 can arrest cell cycle by enhancement of phosphor-p53. JNK enhances JNK-Jun followed by the inhibition of tumor proliferation. Concurrently, allicin induces p38 mitogen that could induce the protein kinase (MAPK) and then increase the expression of Fas binding to Fas ligand (Fas L) and finally activate death pathway through activation of cyt C and caspase-8. Also, the allicin makes caspase-dependent apoptosis through elevating PARP, caspase-3 and caspase-9, which are mediated by enhanced discharging of mitochondria cyt C to the cytosol. The great mitochondrial outer membrane permeability triggers protein kinase A (PKA) and subsequently AIFs associating with its endonucleases G (Endo G) co-activator by directly entering the nucleus of the DNA fragment in a pathway of caspase-independent cell death. Also, allicin induces apoptosis *via* increasing the amounts of free Ca2+, ER stress.

### Allicin as Potential Therapeutic Option in Other Digestive System Cancers

The *in vitro* cytotoxic impacts of allicin have been specified dose-dependently in different mammalian cells (103). According to this, *in situ*-produced allicin activated apoptosis in MIA PaCa-2 (pancreatic cancer) cell line *via* acetylation of Lysine 14 residue at the core histone H3, which its expression has been associated with apoptosis, cell cycle arrest, or differentiation in cancer cells (144).

Chhabria et al. (103) developed a novel method for cancer therapy using targeted delivery of alliinase, which is used to generate *in situ* allicin, and its anticancer effectiveness was specified using the integrated discrete multiple organ co-culture (IdMOC) approach. Based on the data, alliinase established a chemical bond with a monoclonal antibody (mAb), targeting CA19-expressing cells (specific pancreatic cancer marker). Upon addition of alliin, the conjugate CA19-9 mAb–alliinase attached to the targeted MIA PaCa-2 cells followed by allicin production is exposed to the alliinase in the cancerous cells, mediating the apoptosis efficiently in MIA PaCa-2 cell line. The allicin induced DNA fragmentation, expression of caspase-3, expression of p21^WAF1/CIP1^, a CDK inhibitor, cell cycle arrest, GSH depletion, ROS production, and run different epigenetic modifications, which led to the apoptosis induction. By this novel therapeutic strategy in which the combination of targeted agents (alliin as well as alliinase-conjugated antibody) produce allicin, we were able to suppress cancer cell growth and reverse gene silencing as an effective pancreatic cancer therapy (103).

The main constituent of allicin is diallyl trisulfide, which has different medicinal activities, such as antibacterial, antiviral, and antifungal impacts. Since allicin increases the natural killer (NK) cell activity and IL-2 generation as well as promoting T cell proliferation and activity, it can be considered as an immunomodulator (145, 146). It was shown that allicin through its immunomodulatory activity modulated the patterns of cytokine in favor of Th1-type response and improved the efficient cellular response (147). IL-2 is a short glycoprotein generated primarily by CD4+T cell and mediates various biological processes, such as raising the activity of killer cells (NK cells and CD8+T) and stimulating the immune cell for releasing the cytokines (148, 149). Similar to IL-2 structure, rIL-2 (recombinant IL-2) generated by genetic technology can raise T-cell propagation and differentiation, increase the NK cell activity, activate the production of cytotoxic T lymphocyte (CTL), stimulate the generation of tumor-infiltrating lymphocyte (TIL) and lymphokine-activated killer (LAK) cell, activate B-cell proliferation, differentiation, and antibody secretion, and induce the interferon (IFN)-c and other cytokines production (150). At present, rIL-2 is applied as an adjunctive treatment for carcinomas-caused hydrothorax and ascites. Although a short course and low dose of rIL-2 treatment can have considerable clinical benefits, a high dose IL-2 can result in toxicity. Hence, a combination therapy with antiviral agent and rIL-2 could be an approach to treating pancreatic malignancy. Moreover, both rIL-2 and allicin can improve cell-mediated immune responses, which has a significant effect on tumor immunity (117).

Wang et al. (117) examined the effectiveness of combination therapy using allicin and rIL-2 on malignancy of pancreas and its probable immunological mechanism (117). In this study, xenograft pancreatic cancer models in C57/BL6 nude mice classified into four groups were as follows: control (saline), rIL-2-treated, allicin-treated, and rIL-2+allicin combination therapy groups. Subsequent to treatment for 4 weeks, the substantial xenograft growth inhibition and the meaningful prolonged survival time were reported in the combination therapy group. The fluorescence-activated cell sorting (FACS) and terminal deoxynucleotidyl transferase dUTP nick end labeling (TUNEL) tests confirmed a significant increase in the apoptotic cell population in the combination therapy group. Moreover, ELISA depicted that the amounts of NK cell, CD4+T, and CD8+T as well as serum IFN-γ level rose considerably in the combination therapy group. The allicin and rIL-2 combination therapy could suppress the tumor growth and extend the survival time probably *via* NK cell, CD4+T, and CD8+T activation (117).

It was reported that ajoene and related garlic-derived OSCs (DADS and DATS) could arrest the cell cycle at G2/M phase (151, 152). Also, apoptosis has been mediated *via* the cascade of caspase dependent to mitochondrial fashion with the contribution of cytochrome c release, mitochondrial membrane permeabilization (MMP), Bcl-2 (as a antiapoptotic protein) cleavage and caspase-3 activation (52, 152–158). Notably, it has been suggested that (159–161) the antiproliferative property of garlic-derived OSCs might be markedly affected by disulfide bond chemical reactivity, which might thiolate Cys residues in proteins. Accordingly, it has been found that the diallyl trisulfide modifies b-tubulin oxidatively to produce mixed disulfides at Cys residues (Cys-354b and Cys-12b) in a cell-free model (81). In another cell-free environment, the ajoene acted as a covalent suppressor as well as GSH reductase substrate *via* making mixed disulfide at Cys58 on the active site of the enzyme (162).

Kaschula et al. (124) applied a concise four-step synthesis (163) to access end allyl groups substituted-ajoene analogs. Antiproliferation activity of such derivatives library was tested on WHCO1 esophageal cancerous cells, and it was found that the end groups substituted with p-methoxybenzyl (PMB; IC50 = 2.1 μM) is active 12-fold greater than Z-ajoene (159). According to the structure-activity studies, such as the sulfoxide and vinyl disulfide modification, the ajoene pharmacophore is disulfide preventing the growth of WHCO1 cells and stimulating the arrest of G(2)/M cell cycle and apoptotic pathway by caspase-3 stimulation. Moreover, the vinyl group increases the antiproliferative activity a further 8-fold compared to the sulfoxide group (124).

## Limitations of Allicin

Although allicin is short-lived and poorly stable, it can easily cross cell membranes due to its hydrophobic nature. Allicin reacts with free thiol groups rapidly in cellular compartments (164). Alinase converts alliin to allicin at pH 7.0. It can be inactivated by heating or at a pH below 3.5 (165). Therefore, an enteric-coated formulation has been applied to hamper stomach disintegration of many commercial garlic supplements and protect against allinase enzymes (166). A microparticulate formulation, in which alliinase and alliin are individually encapsulated inside microspheres, has been developed for pulmonary administration (167). In a study to examine the allicin bioavailability in 23 types of garlic products in healthy subjects (seven males and six females) 32-h postconsumption, findings demonstrated allicin bioavailability at 36–104% for enteric tablets, 26–109% for garlic powder capsules, 80–111% for non-enteric tablets, 30% for roasted, 16% for boiled, 66% for acid-minced, and 19% for pickled garlic foods (166). Conjugating the alliinase to a monoclonal antibody has been applied as a technique to increase the chemical instability of allicin for a certain marker of pancreatic cancer. These conjugates strongly mediate apoptosis in MIA PaCa-2 cells (103). In addition, encapsulation by liposomes increased the stability of allicin by protecting it against harsh conditions. This technique also reduces the distinct unfavorable aroma (168). Allicin loaded locust bean gum nanoparticle (LBGAN). This system demonstrated protection and stability and improved the allicin pharmacological activity. Furthermore, locust bean gum (LBG) as a natural additive has been shown to be efficient during colon cancer (169). Various stabilized allicin derivatives were synthesized and examined for their activity on multidrug-resistant (MCF-7/Dx) and drug-sensitive (MCF-7) human breast cancer cells. Some of these derivatives were more beneficial than free allicin on starting apoptosis (170).

## Clinical Studies

Despite the significant *in vivo* antitumor effects of allicin on several cancer types, the follow up was not confirmed by the same number of human studies (171). We found only one trial that was recorded on clinicaltrials.gov addressing the efficacy of allicin in cancer (follicular lymphoma NCT00455416), with no published data as of yet. A double-blind, randomized controlled trial comprised of patients with colorectal adenomas has shown that a high dose of AGE led to lower risk of new colorectal adenomas (172). In another randomized multi-interventional trial involving 7.3 years of follow-up, after daily administration of 4 mg steam-distilled garlic oil plus 800 mg garlic extract, precancerous gastric lesion prevalence of gastric cancer occurrence was not affected (173, 174). There are inconsistent clinical trial data from various garlic forms because of the different bioavailability of raw garlic ingredients and the certain garlic supplement formulations (175). In 20 months, AGE the odorous, harsh, and irritating garlic components converted into safe and stable sulfur compounds (176). AGE was demonstrated to reduce the proliferation and prevalence of colorectal cancer (177). Prescribing aged garlic in patients with advanced GI cancer increased NK cell activity but did not change the quality of life (QoL) (178). Furthermore, selenium microdoses or large doses of allitridum were displayed to hamper gastric cancer, particularly in men (179). Even though there is some correlation between higher intake of garlic and onions and lower risk of certain cancer types was approved by epidemiological evidence, the data are restricted and sometimes conflicting. The major epidemiological studies show protective effects of onions and/or garlic against GI cancers. These reports have relied on meta-analyses and systematic reviews (180). The findings of epidemiological studies on colorectal cancer are inconsistent. Some meta-analyses exhibit no decline in the risk of colorectal cancer with higher allium consumption (181). Other case–control studies (2,020 controls and 1,037 cases) found that both garlic and onions were protective against large intestine cancers (182).

## Conclusion

In the last decade, the progression of novel intensive and/or tailored therapies by incorporating targeted therapies and cytotoxic drugs (panitumumab, cetuximab, bevacizumab, regorafenib, and aflibercept for mCRC; ramucirumab and trastuzumab for mGC; and sorafenib for HCC) and accretion of medical treatments with more and more efficient surgical and locoregional approaches meaningfully improved the prognosis of metastatic GI cancer patients (4). Nevertheless, GI cancers are still a prominent reason for cancer death globally (4). Consequently, finding novel therapeutic strategies is vital for the treatment of those patients with cancers. Currently, the combination chemotherapy by the administration of manifold chemotherapeutic agents with diverse biochemical/molecular targets has achieved numerous beneficial effects and ameliorated adverse effects and has been largely applied to different kinds of cancer. There has also been great interest in finding less toxic natural-based substitutes. It was suggested that the plant extracts and herbal-isolated compounds (e.g., curcumin, allicin, resveratrol, and matairesinol) in combination with anticancer drugs have potentially reversed cancer therapy resistance and exerted chemoprotective activities. However, the risks and adverse effects of plant products should be cautiously considered, such as herb–drug interactions. Over the past years, allicin has been broadly used clinically due to the antimicrobial, anti-inflammatory, immunity functions, and cardiovascular protection properties. The anticancer activity of allicin in GI malignancies has emerged *via* inhibiting cell growth and apoptosis-induced cell death.

## Author Contributions

HM and LSH were involved in the conception, design, statistical analysis, and drafting the manuscript. MS, OH, NR, ZN, SM, MR, HP, MMK, MA, and HK contributed to data collection and drafting the manuscript. All authors approved the final version for submission.

## Conflict of Interest

The authors declare that the research was conducted in the absence of any commercial or financial relationships that could be construed as a potential conflict of interest.
